# Tailoring the Magnetic and Electrical Properties of Epoxy Composites Containing Olive-Derived Biochar through Iron Modification

**DOI:** 10.3390/nano13162326

**Published:** 2023-08-13

**Authors:** Erik Piatti, Daniele Torsello, Gaia Gavello, Gianluca Ghigo, Roberto Gerbaldo, Mattia Bartoli, Donatella Duraccio

**Affiliations:** 1 Department of Applied Science and Technology, Politecnico di Torino, C.so Duca degli Abruzzi 24, 10129 Torino, Italy; erik.piatti@polito.it (E.P.); daniele.torsello@polito.it (D.T.); gaia.gavello@polito.it (G.G.); gianluca.ghigo@polito.it (G.G.); roberto.gerbaldo@polito.it (R.G.); 2 Istituto Nazionale di Fisica Nucleare, Sez. Torino, Via P. Giuria 1, 10125 Torino, Italy; 3 Center for Sustainable Future Technologies, Italian Institute of Technology, Via Livorno 60, 10144 Torino, Italy; 4 Consorzio Interuniversitario Nazionale per la Scienza e Tecnologia dei Materiali (INSTM), Via G. Giusti 9, 50121 Firenze, Italy; 5 Institute of Sciences and Technologies for Sustainable Energy and Mobility, National Research Council, Strada delle Cacce 73, 10135 Torino, Italy

**Keywords:** biochar, olive residues, epoxy composites, iron-tailored species, electrical and magnetic properties

## Abstract

The combination of conductive carbon together with magnetic particles is a consolidated strategy to produce cutting-edge fillers for the production of polymer composites able to shield against microwave radiation. In this work, we developed and characterized an iron-tailored biochar obtained from the pyrolysis of olive pruning which was added as filler for the preparation of epoxy composites. The biochar-based composites were obtained by keeping the filler concentration at 10 and 40 wt.%. An extensive characterization was carried out in order to assess the electrical and magnetic properties of the composites containing biochar and iron-tailored biochar. The highest DC electrical conductivity of 59 mS/m was observed in the 40 wt.% iron-tailored biochar-loaded composite, while the reduction of the filler loading led to a drastic reduction in conductivity: 60 μS/m in the 10 wt.%-loaded composite. Ferromagnetic behavior of composites containing iron-tailored biochar is visible in the emerging hysteretic behavior, with a magnetic signal increasing with the filler concentration. Finally, both the complex permittivity (ε’) and the AC conductivity (σ) are enhanced by increasing the BC filler amount in the matrix, regardless of the presence of iron.

## 1. Introduction

The increase in society’s accountability for the preservation and sustainability of natural resource utilization has become one of the major issues of both scientific and regulatory argument [1,2,3,4,5]. Industrial workers and scientific researchers are now more aware of the finite nature of these resources and the detrimental effects of their overexploitation. The research in material science has also focused on advanced solutions based on sustainable materials for cutting-edge applications such as carbon capture [6,7] and conversion [8], energy storage [9,10,11], and electronic applications [12,13]. Furthermore, the production of composites has been oriented to the incorporation of waste- or biomass-derived fillers into polymer matrices. Recycling these materials into composites reduces the burden on landfill sites and the overall environmental impact associated with waste disposal, helping the promotion of a circular economy. Moreover, waste materials are often readily available at lower costs or even for free, as they might be considered as waste byproducts of various industrial processes. Incorporating them into composites can lead to cost savings compared to using expensive virgin fillers. It worth underlining that processing waste- or biomass-derived fillers often requires less energy compared to the extraction and processing of virgin fillers. This can lead to a reduced carbon footprint and contribute to overall energy conservation. Finally, the use of these materials in composites supports the transition towards more sustainable and renewable resources in various industries and also answers to the increasing market demand for sustainable and eco-friendly products. Many industries and consumers are becoming more conscious of the environmental impact of products they use. By incorporating waste- or biomass-derived fillers, companies can meet these demands and comply with environmental regulations.

The resulting composites can exhibit a combination of properties from both the fillers and matrix materials, making them suitable for different applications, including construction, automotive, packaging, and more [14,15,16]. Above all, the production of composites containing tunable carbon-based fillers with high electrical conductivity and magnetic properties represents a field of great interest for sensor development [17,18], EMI shielding, and microwave absorption [19,20]. In fact, it is well-known that that with the rapid expansion of electronic devices and wireless technologies in our daily lives (i.e., mobile, radio, wireless LAN), the concern over electromagnetic interference has become more significant. EMI can disrupt the functioning of electronic devices, communication systems, and even critical infrastructure. The human nervous system may get damaged if exposed to these radiations over an extended period of time [21].

For these reasons, the research interest in creating electromagnetic-interference-shielding materials with superior absorption capability has grown. The most studied solutions are actually based on expensive fillers such as carbon nanotubes [22,23,24,25] or graphene-related materials [26,27,28,29,30]. Nevertheless, alternative conductive carbon fillers have gained great attention, with biochar (BC), i.e., the solid product of biomass pyrolysis being the most promising one considering the high tunability of its combination of properties [31] and the sustainability of its production [32]. The BC electrical properties are strictly related to the production temperature used during the pyrolytic process and to the shape and size of the BC powder. Giorcelli et al. [33] reported a consistent increment of conductivity ranging from the pyrolytic temperature of 400 °C up to 1000 °C, reaching a value of 36 S/m using epoxy resin/BC composites with BC produced from coffee residues. Gabhi et al. [34] evaluated the bulk electrical conductivity of BC monoliths, reporting a maximum value of up to 340 S/m. Furthermore, the authors found an increase in conductivity with the increase in BC carbon content and processing temperature [35]. Interestingly, Giorcelli et al. [36] proved the ability of BC-filled epoxy matrixes to shield electromagnetic radiation with a performance comparable to that of carbon-nanotube materials. Accordingly, the production of BC-containing composites with good high-frequency electromagnetic absorption has gained a great interest for both polymeric [37] and construction [38,39,40] materials applications. Additionally, BC could be easily tailored with magnetic particles for the realization of magnetic powders used for environmental remediation [41,42] or for the production of multifunctional composites [17,43,44]. In general, the addition of magnetic metals [27] has a good impact on the composite’s final properties and can increase the effectiveness of MW shielding. The metallic species employed for the shielding against MW are often difficult to prepare and incredibly expensive complex metal oxides [45,46] or iron-based nanoparticles [47,48]. In the present research work, we produced an iron-tailored olive BC (Fe@BC) by a simple carbothermal route. This means that reducing agents such as carbon sources are used to reduce substances (such as metal oxides). Temperatures of several hundred degrees Celsius are typically used for these chemical processes that are often used to produce the elemental forms of several elements. Carbothermal reduction techniques provide certain unmatched benefits, including ease, a cheap cost, and remarkable reproducibility for industrial uses.

It worth underlining that olive residues have been chosen as the waste stream because they are particularly available in the south of Europe and are currently disposed of by simply being burnt. These residues are generated in significant quantities from olive oil production and olive processing industries and include olive pits, olive pomace, and olive tree prunings. The abundance and availability of olive residues make them a reliable and sustainable source for the production of high-added-value materials and can represent a solid choice to promote more friendly waste management. The innovative aspect of this work is that Fe@BC is used for the first time in the preparation of magnetic conductive epoxy composites that were extensively characterized both under DC and AC regimes and through magnetic hysteresis cycle measurements. Fe@BCs were characterized by field-emission scanning electron microscopy (FESEM) Raman and XPS spectroscopy.

## 2. Materials and Methods

### 2.1. Materials

Olive residues were collected in Imperia (Liguria, Italy) and dried at 105 °C for 72 h before their use. Two-component BFA diglycidyl resin was purchased from CORES (Cores epoxy resin, LPL). Iron nitrate heptahydrate (>99%) was provided by Sigma-Aldrich and used without further purification.

### 2.2. Methods

#### 2.2.1. Fe@BC Production

Olive residues were cut into cylindrical pieces with an average size of 3 × 15 cm^2^ and pyrolyzed in a tubular furnace (Carbolite TZF 12/65/550, Neuhausen, Germany) by using nitrogen atmosphere and a heating rate of 10 °C/min for reaching and keeping the systems at 800 °C for 30 min. The produced BC was pulverized using a TURBULA^®^ mixer T 2 F (Muttenz 1Switzerland) for 2 h and then mixed with iron nitrate heptahydrate (weight ratio of 2:1) by suspending the species in water and drying them at 130 °C for 24 h. The resulting powder was ground and pyrolyzed again in a tubular furnace (Carbolite TZF 12/65/550) in nitrogen atmosphere using a heating rate of 10 °C/min reaching and keeping the systems at 550 °C for 30 min. Fe@BC was recovered and used without any further purification.

#### 2.2.2. Fe@BC-Containing Epoxy Composites Preparation

Fe@BCs were dispersed into resin monomer by using a tip ultrasonicator apparatus (Sonics Vibra-cell) for 15 min, pulsing the ultrasounds with on/off cycles of 20/10 s to avoid an excessive temperature increase. After adding the curing agent, the resulting solution was additionally treated for 2 min, poured into a silicon mould, and left there for 16 h at 24 °C. The thermal curing was completed in a ventilated oven (I.S.C.O. Srl “The scientific manufacturer”, Venezia, Italy) at 80 °C for 4 h. The amount of Fe@BC into the composites was 10 and 40 wt.%, thus including the common bottom and high ranges adopted in the literature for the production of conductive biochar-based epoxy materials. Composites comprising BC without Fe tailoring were also synthesised as a reference for electrical and magnetic characterizations.

#### 2.2.3. Characterization of Fe@BC and Composites

The Raman spectrum of Fe@BC powder was collected using a Renishaw inVia (H43662 model, Gloucestershire, UK) equipped with a laser line with a wavelength of 514 nm and a 50× objective. Raman spectra were recorded in the range from 150 cm^−1^ to 3500 cm^−1^ and the region between 1000 and 2000 cm^−1^ was analyzed with a homemade software compiled in Matlab^®^ (version R2020a) following a procedure reported by Tagliaferro et al. [49].

Fe@BC morphology was observed in a field-emission scanning electron microscope (FE-SEM, Zeiss SupraTM40, Oberkochen, Germany). The instrument was equipped with an energy-dispersive X-ray detector (EDX, Oxford Inca Energy 450, Oberkochen, Germany) employed for a compositional evaluation of Fe@BC.

Fe@BC chemical functions were investigated by using an XPS spectrometer (PHI 5000 Versaprobe Physical Electronics, Chanhassen, MN, USA) equipped with a monochromatic Al K-alpha X-ray source with 1486.6 eV energy using 5 kV voltage, and 1 mA anode current.

The DC electrical conductivity of the composites was measured at room temperature (295 K) [50] by electrically contacting the composite samples in the four-point van der Pauw configuration by using thin gold wires and conducting silver paste (RS Components, Sesto San Giovanni, Italy). A current of 1 μA was sourced between two adjacent contacts with a B2912 source measure unit (Keysight Technologies, Santa Rosa, CA, USA), for each van der Pauw configuration. The voltage drop occurring across the opposite two contacts was measured with a 34,420 nanovoltmeter (Keysight Technologies, Santa Rosa, CA, USA). The conductivity was then calculated as $\sigma =R_{s}^{-1}t^{-1}$, where $R_{s}$ is the sheet resistance obtained by solving the van der Pauw equation, and t is the thickness of the material. By loading the samples in the high-vacuum chamber of an ST-403 pulse-tube cryocooler (Cryomech, Syracuse, NY, USA), cooling the system to the base temperature of 2.7 K, and then quasi-statically warming the samples to 295 K, the temperature dependence of σ was measured in accordance with the method reported in literature [51]. By inverting the sourced current flow within each resistance measurement, thermoelectric voltages were eliminated. Due to an imperfect thermal coupling between the sample and the thermometer, data points below 20 K were excluded.

The real parts of the complex permittivity (ε’) and the conductivity (σ) of the samples were measured in the GHz range by means of a cylindrical coaxial cell (EpsiMu toolkit [51]), which contains the composite as a dielectric spacer between the inner and outer conductors, whose diameters are 3 mm and 7 mm, respectively. The cell was linked to standard connectors, keeping the characteristic impedance at 50 Ω, thus avoiding mismatch and energy loss. The cell was connected to a Rohde Schwarz ZVK Vector Network Analyzer that was suitably calibrated, and measurements were analyzed with a two-port transmission line technique. By de-embedding and using the Nicolson–Ross–Weir transmission/reflection algorithm, the sample’s electromagnetic characteristics were measured.

An electromagnet-equipped DC magnetometer/AC susceptometer (Lakeshore 7225) was used to study the magnetic characteristics in quasi-static conditions at an ambient temperature. To specifically determine the magnetic behavior, magnetic hysteresis cycle measurements on the composites up to 30 kA/m were performed. Considering that the signal from the epoxy matrix was hardly detectable and that the composition’s weight and filler content were known from the preparation, it was possible to characterize the magnetic properties of the filler alone.

## 3. Results

### 3.1. Characterization of Fe@BC

**Figure 1:**
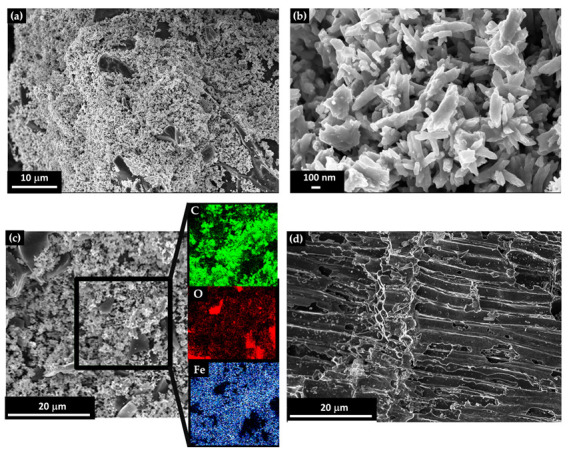
FESEM images of (**a**) Fe@BC with a focus on (**b**) sub-micrometric iron particles, (**c**) an EDX map for elemental analysis in false colors (green for carbon, red for oxygen, and blue for iron), and (**d**) pristine BC.

The morphology and chemical composition of the Fe@BC powder was carried out through FESEM-EDX analysis, and the results are summarized in Figure 1 and Table 1.

As shown in Figure 1a, the tailoring process of the olive BC is quite effective compared with that of the classical porous surface of neat olive BC. In fact, the channeled surface of BC (Figure 1d) is not more observable due to the effective covering of iron-based particles. The addition of iron nitrate promotes a uniform covering of the BC surface with the production of rod-like particles with an average length of 200 nm and 50 nm in diameter (Figure 1b). This rod-like morphology, that depends on the carbothermal reaction conditions, is reported for the first time as a metallic tailoring biochar surface. It could be ascribed to the non-total conversion of iron nitrate to metallic nanoparticles that are already described in literature as spherical for this procedure. The morphology is similar to that of the rhombic ferrite species as observed by Cheng et al. [52]. The EDX elemental map reported in Figure 1c shows a good covering of the carbon surface by the iron species, with few exceptions corresponding to small BC flakes. Moreover, the oxygen and iron distributions do not appear to coincide in Figure 1c (red and blue colors), and this is probably due to the presence of a small excess of iron (III) oxide. As summarized in Table 1, oxygen species are associated with both carbon and iron and represented up to 21 wt.%, while the carbon and iron species represented 27 and 52 wt.%, respectively.

**Figure 2:**
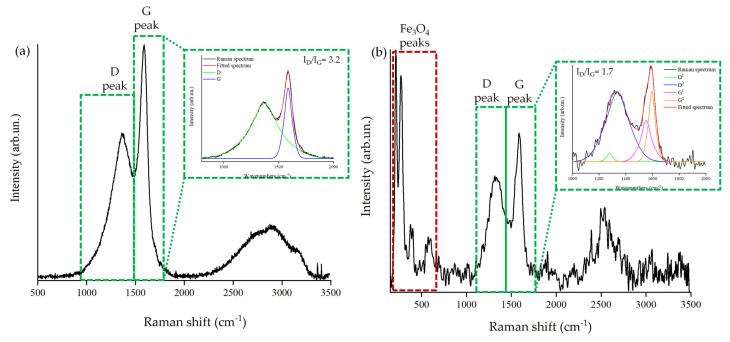
Raman analysis of (**a**) BC and (**b**) Fe@BC between 150 and 3500 cm^−1^. The fitting of both G and D peaks in the region between 1000 to 2000 cm^−1^ [40] is reported in the green dotted square [49].

The Raman study reported in Figure 2 offers more information on the composition of Fe@BC.

**Figure 3:**
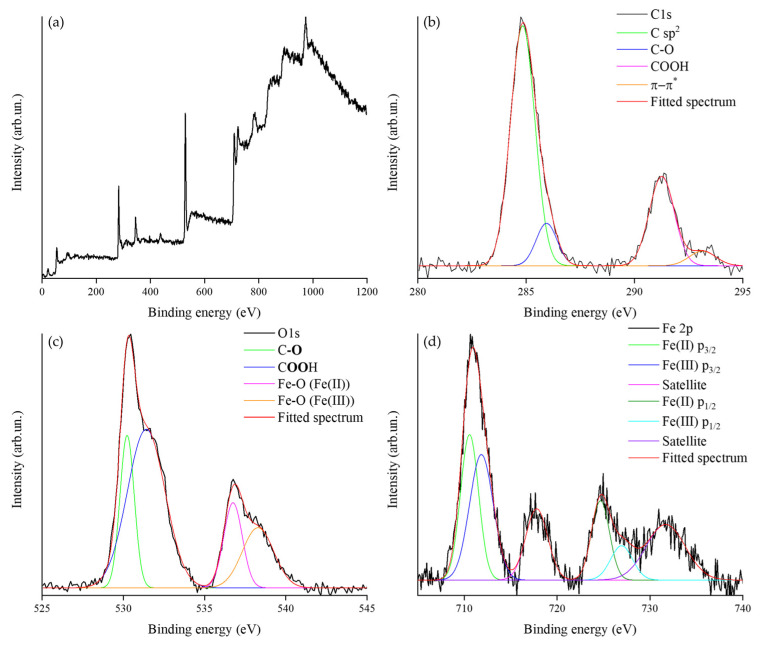
XPS analysis of Fe@BC showing (**a**) the survey and fitted (**b**) C 1s, (**c**) O 1s, and (**d**) Fe 2p spectra.

As shown in Figure 2a, BC shows a very high I_D_/I_G_ ratio of up to 3.2, suggesting the presence of a disorganized carbonaceous structure [53], while Fe@BC powder shows an I_D_/I_G_ ratio of up to 1.7, implying the reduction of less-crystalline carbon. This is in agreement with the carbothermal reduction process that consumes the less-crystalline carbon due to its higher reactivity. Furthermore, the presence of peaks at 215, 272, 378 and 585 cm^−1^ supports the presence of Fe_3_O_4_ iron species on BC as reported by Yew et al. [54]. The formation of ferrite is promoted by a partial reduction of Fe(III) induced by the oxidation of the carbon matrix [55,56,57]. The investigation of the surface chemical states of carbon, oxygen, and iron was carried out by using XPS analysis, reported in Figure 3 and summarized in Table 2. This investigation also allows us to check the massive formation of mixtures of metal oxides and metallic species that can occur during the carbothermal process as a consequence of the selected operative temperature [58]. 

The survey spectrum (Figure 3a) shows that no significant inorganic contaminants were present in the samples such as inorganic residues from the original biomass. The C 1s spectrum shows the massive presence of C sp^2^ (284.8 eV) at up to 65%, together with hydroxyl (285.9 eV) and carboxylic (291.3 eV) functions at up to 10 and 25%, respectively. Interestingly, carbonyl functions were not detected, suggesting the removal of highly reactive carbon–oxygen sites (particularly on the material edges) during the pyrolysis process. This result is in agreement with the Lerf model for the oxygen function distribution on oxidized carbon [59]. Accordingly, the O 1s spectrum shows only the signal due to hydroxyl (530.2 eV) and carboxylic (531.4 eV) species with abundances of up to 19 and 52%, respectively. Furthermore, the signal of Fe-O indicates the simultaneous presence of Fe(II)-O (536.8 eV) and Fe(III)-O (538 eV) with concentrations of up to 14 and 15%, respectively [60]. Similarly, the Fe 2p signal suggests the presence of both Fe(II) (Fe 2p_1/2_ at 710.7 eV, Fe 2p_3/2_ at 724.7 eV) and Fe (III) (Fe 2p_1/2_ at 711.9 eV, Fe 2p_3/2_ at 726.9 eV) with abundances of 47 and 53%, respectively. Fe(0) was not detected, supporting the effectiveness of carbothermal reduction control, with the small excess of Fe(III) reasonably due the small excess of Fe_2_O_3_ formed during the process.

### 3.2. Characterization of Fe@BC Composites

#### 3.2.1. DC and Low-Temperature Electrical Characterization

The DC electrical conductivity of the composites containing different filler amounts was first measured at ambient conditions. The composite containing 40 wt.% BC without Fe particles exhibited a conductivity of 16 mS/m. This conductivity value is indeed much lower than the maximum ones reported in the cited works by Giorcelli et al. [33] and Gabhi et al. [34]. This is because the conductivity of biochar (and its composites) is critically dependent on the pyrolysis conditions, which in turn determine the degree of carbonization and of oxygen–carbon and hydrogen–carbon atomic ratios, on which the final conductivity depends in non-trivial ways. In general, higher pyrolysis temperatures and longer pyrolysis times result in more conductive biochar (and related composites). However, the conductivity found in this work is comparable with our previous reports on hemp-derived BC [17] and coffee-derived BC [33] at comparable pyrolysis conditions. Among the composites containing Fe@BC, a conductivity of 59 mS/m was observed in the 40 wt.%-loaded composite, while the reduction in the filler loading caused a drastic reduction in conductivity: 60 μS/m in the 10 wt.%-loaded composites. This result suggests that these latter filler loadings are below the percolation threshold for the composite.

**Figure 4:**
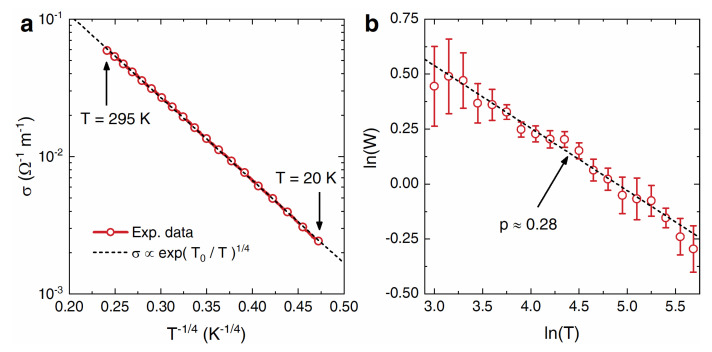
(**a**) DC electrical conductivity σ of the 40 wt.%-loaded composite vs. $T^{-1/4}$ between 295 K and 20 K (in semilogarithmic scale). The ideal scaling expected for 3D Mott variable-range-hopping conduction is represented by the black line. (**b**) W=d(lnσ)/d(lnT), that is the reduced activation energy vs. the temperature in double logarithmic scale. The black line is a linear fit to the data in the entire temperature range and has a negative slope p≈0.28 that is consistent with the 3D Mott variable-range-hopping value of p=1/4.

The relatively large conductivity of the 40 wt.%-loaded composite allows for a reliable investigation of its electric transport mechanism by measuring the temperature dependence of the conductivity down to low temperatures. The black dashed line in Figure 4a emphasized how σ strongly decreases when the temperature is decreased from 295 to 20 K, and that is scaled well, as $\sigma \left(T\right)=\sigma_{0}\exp{\left(T_{0}/T\right)}^{1/4}$ when $T_{0}\approx 37,000$ K. This behavior is typical of insulating materials, where electric transport occurs via three-dimensional (3D) Mott-type variable-range hopping (VRH), according to the theory of the insulator-to-metal transition [61], and is frequently seen in electrically conducting composite materials [62], such as ceramic–metal composites (cemets) [63], carbon-based composites and metal clusters [64], and, more recently, 2D material networks [65]. In other words, this means that the electric conduction in the epoxy composites does not occur by diffusive transport over extended states (as in a typical metal), but by thermally activated hopping between localized states. Indeed, most composite materials which are electrically conductive at room temperature [62] do exhibit a hopping transport mechanism and therefore become unable to support the flow of electrical currents when cooled down close to the absolute zero, unlike a metal which retains a finite conductivity in such conditions instead.

Figure 4b, which reports the Zabrodskii analysis [66] of the reduced activation energy W=d(lnσ)/d(lnT), confirms this picture since in the entire considered temperature range, lnW linearly drops off with the increase of lnT and with a negative slope p≈0.28. This value is consistent with the one expected for 3D Mott VRH (p=1/4) and rules out 2D Mott VRH (p=1/3), Efros–Shklovskii VRH (p=1/2), and nearest-neighbor hopping (p=1) as possible alternative conduction mechanisms.

#### 3.2.2. Magnetic Characterization

**Figure 5:**
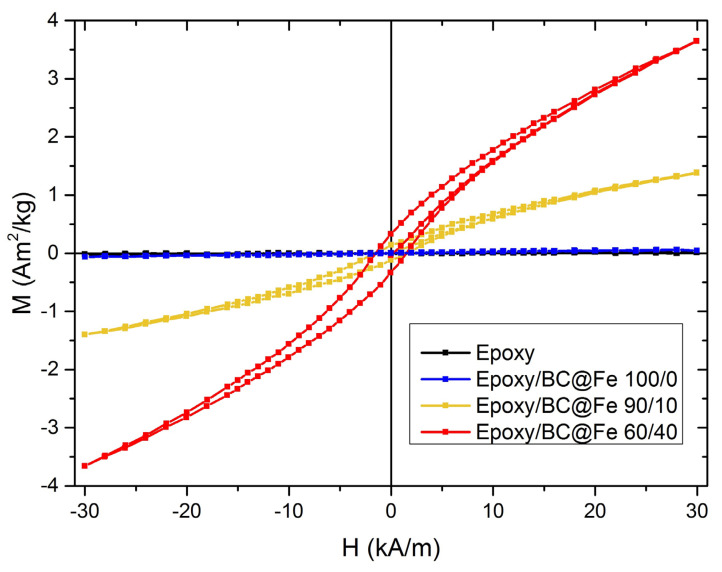
Cycles of hysteresis for the unfilled epoxy and composites up to 30 kA/m.

The cycles of hysteresis, with applied fields of up to 30 kA/m, are shown in Figure 5. The pure epoxy matrix (black curve) and the sample containing BC without Fe particles (blue curve) do not show any magnetic behavior. Conversely, the ferromagnetic behavior of composites containing Fe@BC is perfectly visible in the emerging hysteretic behavior. Moreover, the magnetic signal increases with the increase of the filler concentration. This expected behavior is interesting for a broad range of applications where magnetic composites are desirable.

#### 3.2.3. Electrical Characterization at High Frequency

**Figure 6:**
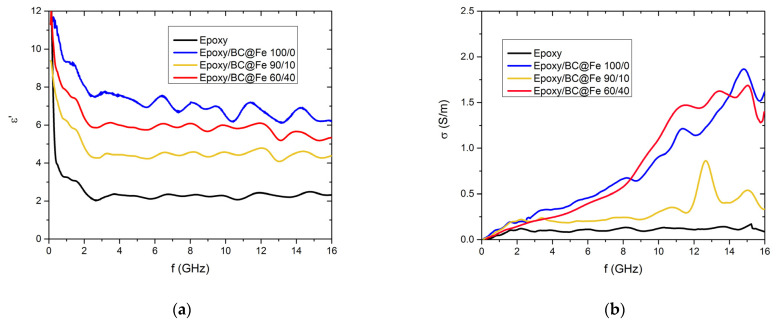
(**a**): the real part of the complex permittivity (ε’). (**b**): the conductivity (σ) vs. frequency.

In Figure 6, the real part of the complex permittivity (ε’) and the conductivity (σ) are presented vs. the frequencies (f) for the pure epoxy matrix (black curve), the sample containing BC without Fe particles (blue curve), and all the composites containing Fe@BC (yellow and red curves).

Clearly, both the complex permittivity (ε’) and the conductivity (σ) are enhanced by introducing BC in the matrix regardless of the presence of Fe, which mostly affects the magnetic properties. Moreover, in the high-frequency regime, the conductivity is not affected by the network of the filler (no percolation threshold needs to be overcome to obtain high conductivity values since the local conductivity is probed), and this results in a direct increase of σ to the same order of magnitude in all the samples. It is worth noting that the filler weight percentage is the same for all the samples, but the amount of BC in each filler is different. Moreover, the electronic properties of the BC in each filler are also different due to the higher graphitization of C promoted by the carbothermal reduction process [67]. For this reason, a non-trivial behavior of ε’ and σ with increasing BC emerges: their values do not simply increase with increasing amounts of either BC or Fe in the epoxy matrix. Interestingly, it is not strictly necessary to maximize the conductivity of BC materials [40,68,69] to improve the MW properties, as shown by several works in the literature [36,70]. As discussed by Natalio et al. [71], BC produced at 800 °C can be as effective a filler for MW shielding as the one produced at a higher temperature due to the complex model that described the EMI shielding [72,73]. Accordingly, a reasonable temperature treatment trade-off together with a magnetic tailoring represents a solid choice to produce an effective filler for EMI shielding applications.

## 4. Conclusions

The production of new iron-tailored BC materials (Fe@BC) has been reported by using low-temperature carbothermal synthesis. BC was obtained by the pyrolysis of olive residues generated in significant quantities from olive oil production and olive processing industries. The biochar-based composites were obtained by keeping the filler concentration at 10 and 40 wt.%. The Fe@BC powder showed a peculiar morphology with ferrite rod-like submicrometric particles covering the carbon surface in a very homogenous way. The powder characterization clearly showed the absence of any trace of metallic iron and a small excess of Fe(III), probably due to the overoxidation on the particle edges and to the no-total conversion of iron nitrate to metallic nanoparticles. The morphology is similar to that of the rhombic ferrite species. The combination of electrical and magnetic measurements showed remarkable conductivity and a pure ferromagnetic behavior, with the magnetic signal increasing with the filler concentration. The iron tailoring procedure significantly affected the conductivity of BC, improving the graphitization and achieving the same results using the neat BC, proving the degradation of a more reactive and disorganized carbon domain. All the results clearly suggest that Fe@BC powder represents an interesting material for the development of advanced multi-property epoxy composites for use in several technological fields.

## Figures and Tables

**Table 1:** Elemental composition of Fe@BC calculated through EDX analysis.

Element	wt.(%)
Carbon	27
Oxygen	21
Iron	52

**Table 2:** Chemical species distribution calculated from the fitted XPS spectra.

Chemical Species (atomic %)								
Carbon			Oxygen				Iron	
*C sp^2^*	***C*** *-O*	***C*** *OOH*	*C-**O***	*C**OO**H*	*Fe-O (Fe(II))*	*Fe-O (Fe(III))*	*Fe(II)*	*Fe(III)*
65	10	25	19	52	14	16	47	53
